# Knowledge and Preparedness of Physicians in Relation to Anaphylaxis at Primary Healthcare Centers in Al-Qassim, Saudi Arabia

**DOI:** 10.7759/cureus.57153

**Published:** 2024-03-28

**Authors:** Haifa N Alsaleem, Ahmed S Almuzaini, Fai N Aldakheel, Raghad Almuhaisni, Nasser A Alsharekh, Meshal K Alharkan, Lama N Aldakhil, Abdullah s Aljudayi, Khalid A Alkhalifah, Aqeel F Altuwaiyan, Ali Y Alsaleemi

**Affiliations:** 1 Medicine, College of Medicine, Qassim University, Buraydah, SAU; 2 Allergy and Immunology, College of Medicine, Qassim University, Buraydah, SAU

**Keywords:** life threatening anaphylaxis, healthcare, physician, practice, knowledge, anaphylaxis

## Abstract

Background

Anaphylaxis is a significant, often fatal, systemic allergic reaction with a rapid start that may affect the respiratory and/or circulatory systems; for patients to survive, emergency management must be done properly. When anaphylaxis is confirmed or highly suspected, epinephrine should be injected intramuscularly. This study aimed to assess the preparedness of primary healthcare physicians for anaphylaxis in terms of recognition and management of this condition in Al-Qassim, Saudi Arabia.

Methods

This cross-sectional study was carried out among primary healthcare physicians in the Qassim region of Saudi Arabia. A self-administered questionnaire was distributed among the targeted physicians. The questionnaire comprised sociodemographic characteristics, general awareness and management of anaphylaxis, and a 10-item questionnaire to assess physicians' knowledge of anaphylaxis.

Results

Out of 121 primary healthcare physicians, 61.2% were male, and 47.9% were aged between 25 and 35 years. Nearly all physicians (97.5%) believed that anaphylaxis is a life-threatening situation. The overall mean knowledge score was 4.74 out of 10 points. Nearly half (48.8%) were categorized as having poor knowledge levels, 43% as moderate, and only 8.3% as having good knowledge. Being specialists/consultants and being trained in managing anaphylaxis were the factors associated with increased knowledge.

Conclusion

The knowledge of primary care physicians regarding anaphylaxis was deficient. However, increased knowledge of anaphylaxis was seen more frequently among consultants or specialists who attended training for managing anaphylaxis cases. A multi-center study involving a bigger sample size is needed to establish physicians' knowledge of anaphylaxis.

## Introduction

Anaphylaxis is most commonly described as a significant, often fatal systemic allergic reaction with a rapid onset that may affect the respiratory and/or circulatory systems [1]. Since anaphylaxis is a multisystemic body reaction, the signs and symptoms vary from person to person and the type of allergens one is exposed to. For instance, pulmonary symptoms particular to children are typically more common than cardiovascular and cutaneous indicators related to adults. The most frequent clinical symptoms of anaphylaxis include urticaria, angioedema, erythema, itching, trouble breathing, swelling of the tongue, and death from cardiovascular collapse or pulmonary obstruction [2]. However, anaphylaxis is usually difficult to detect due to the wide variability in signs and symptoms [3]. Three studies showed that primary care physicians and pediatricians have inadequate knowledge when it comes to recognizing the signs and symptoms of anaphylaxis or properly diagnosing it [4-6].

Foods are by far the most frequent causes of anaphylaxis in children, and include peanut, tree nuts, milk, eggs, crustacean shellfish, and finned fish. Anaphylaxis can also be brought on by certain allergies, medicines, and insect bites [7]. For patients to survive, emergency management must be done properly. The prognosis is frequently disregarded by the patient or physician, which frequently results in an undesirable consequence [8].

Various resources offer management guidelines for anaphylaxis. However, the necessity of using epinephrine cannot be questioned [9]. As soon as anaphylaxis is confirmed or highly suspected, epinephrine should be intramuscularly injected in the mid-anterolateral aspect of the thigh at a dose of 0.01 mg/kg of a 1:1,000 (1 mg/mL) solution, up to a maximum of 0.5 mg in adults (0.3 mg in children) [10]. Unfortunately, there is a lack of knowledge of the correct dose for auto-injector administration among physicians. Few physicians are aware of the two available doses of epinephrine auto-injectors [11-13]. Lack of knowledge about appropriate routes of administration of epinephrine is also an identified gap [13-16].

The prevention and management of anaphylaxis depend heavily on primary care physicians who are the first point of contact for patients [3]. Healthcare professionals must understand the urgency of an anaphylactic shock and that it could be lethal if treatment is postponed for even 15 minutes [17]. Numerous studies done in the past show that there is uncertainty among medical professionals when choosing the first-line medicine for treating the emergency situation, and also a lack of knowledge regarding the dose and method of administration of adrenaline [18].

A study was carried out in Madina, Saudi Arabia, with 112 medical professionals as participants, where participants' knowledge was assessed through 20 questions. The mean and median scores were both 60%. This means that 50% of those who took the test scored less than 60%. The majority of participants received scores ranging from 41% to 60% [19]. Another study from Al-Qassim, Saudi Arabia, focusing on the knowledge and attitude of teachers in relation to anaphylaxis showed that most teachers had a low level of knowledge (85.3%) regarding anaphylaxis [20]. However, there is limited information in the literature about the knowledge of anaphylaxis management in Al-Qassim. The aim of this study was to assess the preparedness of primary healthcare physicians in relation to recognition and management of this condition by them in Al-Qassim, Saudi Arabia.

## Materials and methods

Study design and sampling technique

This study was designed as an observational cross-sectional study conducted at primary healthcare facilities of the Qassim region. The sample size for this study was calculated using the Statistical Book of 2022 provided by the Ministry of Health, Saudi Arabia. The Qassim region has 152 primary healthcare centers, and with one physician selected from each center as a representative of the population, we used a sample calculator to reach the sample size. With a population of 152, a confidence level of 95%, a margin of error of 5%, and the population proportion of 50%, the results showed a sample size of 110 physicians at primary healthcare centers in the Al-Qassim region. We distributed the questionnaire among 121 physicians in order to achieve a more accurate representation of the population, as the minimum required sample size was 110. The response rate was 100%.

Data was collected from physicians at randomly selected primary healthcare centers in the Qassim region after obtaining their consent. This was done via a self-administered questionnaire consisting of two sections. The first section included sociodemographic details of the healthcare providers and the second section was about anaphylaxis awareness. The inclusion criteria included physicians working at primary healthcare centers in the Qassim region who agreed to participate. Non-physician practitioners were excluded from the study. The study was approved by the Regional Research Ethics Committee, Ministry of Health, Al-Qassim (no. H-04-Q-001).

Questionnaire criteria

The knowledge of anaphylaxis was assessed using a 10-item questionnaire; the correct answer for each question was identified and coded as 1, while the incorrect answer was coded as 0. The total knowledge score was calculated by adding all 10 item scores. A score ranging from 0 to 10 points was achieved, with the greater the score, the greater the knowledge of anaphylaxis. Using 50% and 75% as cutoff points to determine the knowledge levels, physicians were considered to have poor knowledge if the total score was <50%; 50% to 75% was considered moderate and above 75% was considered a good knowledge level [21].

Statistical analysis

Categorical variables were defined as numbers and percentages (%), while continuous variables were computed and expressed as means and standard deviations. The differences in knowledge scores in relation to the sociodemographic characteristics and general awareness about anaphylaxis were evaluated using the Mann-Whitney Z-test and Kruskal-Wallis H-test. Statistical collinearity was evaluated using the Shapiro-Wilk test as well as the Kolmogorov-Smirnov test. The knowledge score followed the non-normal distribution. Therefore, the non-parametric test was applied. Multiple mean comparisons of knowledge scores in terms of the professional status group were also performed for the significant differences between each professional group. A p-value of less than 0.05 was considered statistically significant. All statistical data were analyzed using IBM SPSS Statistics, version 26 (IBM Corp., Armonk, NY).

## Results

A total of 121 physicians were enrolled in this study. Table 1 describes the sociodemographic characteristics of physicians: 47.9% were aged between 25 and 35 years, with males being dominant (61.2%). Nearly half were working in Buraydah (47.1%). The most common professional group was of general practitioners (54.5%). In addition, 50.4% had at least less than five years of working experience.

**Table 1:** Sociodemographic characteristics of physicians (n=121)

Study variables	N (%)
Age group	
<25 years	02 (01.7%)
25–35 years	58 (47.9%)
36–45 years	45 (37.2%)
>45 years	16 (13.2%)
Gender	
Male	74 (61.2%)
Female	47 (38.8%)
City of work	
Buraydah	57 (47.1%)
Unaizah	25 (20.7%)
Ar Rass	14 (11.6%)
Al-Bukayriyah	08 (06.6%)
Al-Asyah	17 (14.0%)
Professional status	
General practitioner	66 (54.5%)
Resident	21 (17.4%)
Specialist	24 (19.8%)
Consultant	10 (08.3%)
Years of experience	
<5 years	61 (50.4%)
5–10 years	31 (25.6%)
>10 years	29 (24.0%)

When examining the general awareness of physicians regarding anaphylaxis, it was observed that most of the physicians (97.5%) believed that allergy threatens human life (Table 2). Physicians who attended a course or were trained in the management of anaphylaxis constituted 38.8% and 66.1%, respectively, of the sample size. A total of 53.7% had encountered a patient with anaphylaxis. In total, 36.4% physicians were aware of the nearest allergy clinic in their area, while 83.5% kept epinephrine drugs in their department. Nearly three-fourths (73.6% and 71.1%, respectively) were aware that their primary healthcare center had protocols and educational materials for managing anaphylaxis. Physicians who had heard of epinephrine auto-injectors constituted 71.1% of the total sample, and the availability of this device at their primary healthcare center was mentioned by 19.8%. Most physicians (95.9%) indicated needing more training/courses on anaphylaxis management. Additionally, 44.6% had heard of Saudi Allergy, Asthma and Immunology Society (SAAIS).

**Table 2:** General awareness about anaphylaxis and its management (n=121)

Questions	N (%)
Do you think that allergy can be life-threatening?	
Yes	118 (97.5%)
No	03 (02.5%)
Have you ever attended a course on the management of anaphylaxis?	
Yes	47 (38.8%)
No	74 (61.2%)
Have you ever been trained in the management of anaphylaxis?	
Yes	80 (66.1%)
No	41 (33.9%)
Have you ever met a patient with anaphylaxis?	
Yes	65 (53.7%)
No	56 (46.3%)
Do you know where the nearest allergy clinic is in your area?	
Yes	44 (36.4%)
No	77 (63.6%)
Do you keep epinephrine drugs in your department?	
Yes	101 (83.5%)
No	20 (16.5%)
Does your primary healthcare center have a protocol to treat anaphylaxis?	
Yes	89 (73.6%)
No	32 (26.4%)
Do you have an action plan and educational materials available in your primary healthcare center for patients/parents to treat anaphylaxis at home?	
Yes	47 (71.1%)
No	36 (29.8%)
I don't know	38 (31.4%)
Have you ever heard about epinephrine (Epipen®) auto-injector?	
Yes	86 (71.1%)
No	35 (28.9%)
Is epinephrine auto-injector available for outpatient prescriptions in your primary healthcare center?	
Yes	24 (19.8%)
No	66 (54.5%)
I don't know	31 (25.6%)
Do you feel the need for more training/courses in anaphylaxis management?	
Yes	116 (95.9%)
No	05 (04.1%)
Have you heard of Saudi Allergy, Asthma and Immunology Society (SAAIS)?	
Yes	54 (44.6%)
No	67 (55.4%)

**Figure 1:**
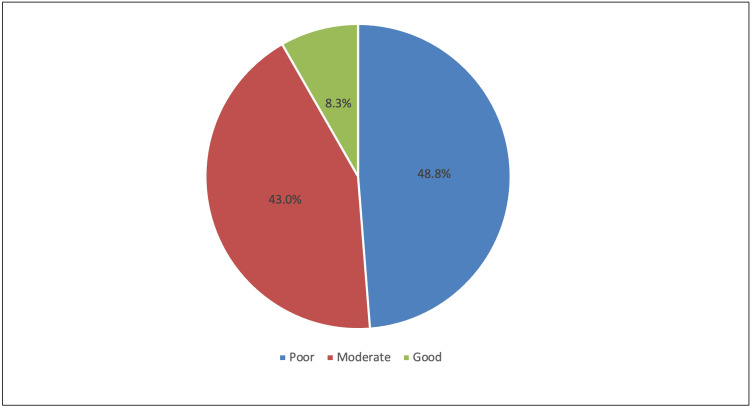
Level of knowledge toward anaphylaxis

Regarding the assessment of a physicians' knowledge about prophylaxis, physicians were found to have poor knowledge in most of the 10 knowledge items (Table 3). Poor knowledge was notably seen when asked about the right appropriate dose of the epinephrine dose as an auto-injector for adults (correct answer, 19.8%), the best position for anaphylactic patients (correct answer, 27.3%), recommended intravenous dose of epinephrine (correct answer, 31.4%) and the duration for follow-up after reaction in anaphylaxis patients (correct answer, 33.9%). Only in reply to the question about the first-line medication in treating a patient with anaphylaxis, our physicians showed excellent knowledge (correct answer, 90.1%). The overall mean knowledge score was 4.74 (SD 1.99), with 48.8%, 43%, and 8.3% showing poor, moderate, and good knowledge levels, respectively (see also, Figure 1).

**Table 3:** Assessment of knowledge about anaphylaxis (n=121)

Question	N (%)
What is the clinical criterion for diagnosing anaphylaxis? [Acute onset of an illness with involvement of skin and at least one of the following: respiratory or cardiovascular symptoms]	62 (51.2%)
What is the best position for anaphylactic patients? [Recumbent position, with leg elevation]	33 (27.3%)
Which is the first-line medication in the treatment of a subject with anaphylaxis? [Epinephrine]	109 (90.1%)
What is the interval of re-administration of epinephrine? [5 minutes]	64 (52.9%)
What is the recommended route of epinephrine administration as the first line of action in anaphylaxis? [Intramuscular]	88 (72.7%)
What is the appropriate intramuscular dose of epinephrine? [0.3 mg/kg of a 1/1000 solution of epinephrine]	60 (49.6%)
What is the recommended location for administering epinephrine as intramuscular? [Vastus lateralis (mid-anterolateral thigh)]	54 (44.6%)
What is the recommended intravenous dose of epinephrine? [1/10,000 epinephrine 0.1 mg/mL]	38 (31.4%)
How long should the patient with anaphylaxis be followed up after the reaction? [6-8 hours]	41 (33.9%)
What is the appropriate dose of epinephrine as an auto-injector for adults? [300 micrograms]	24 (19.8%)
Total knowledge score (mean ± SD)	4.74 ± 1.99
Level of knowledge	
Poor	59 (48.8%)
Moderate	52 (43.0%)
Good	10 (08.3%)

When measuring the differences in knowledge scores according to the sociodemographic characteristics and the general awareness toward anaphylaxis (Table 4), it was found that a higher knowledge score was more associated with being a specialist/consultant (H=0.7.961; p=0.019) and being trained in the management of prophylaxis (Z=2.369; p=0.018). No significant differences were observed between the knowledge scores among the rest of the demographic variables and the general awareness about prophylaxis (all p>0.05).

**Table 4:** Differences in knowledge scores in relation to the sociodemographic characteristics and general awareness about anaphylaxis (n=121) ^§^p-value has been calculated using the Mann-Whitney Z-test. ^‡^p-value has been calculated using the Kruskal-Wallis H-test. **Significant at p<0.05 level.

Factor	Knowledge score (10), mean ± SD	Z/H-test	p-value^§^
Age group			
≤35 years	4.97 ± 1.84	1.099	0.272
>35 years	4.51 ± 2.13		
Gender			
Male	4.86 ± 1.96	0.942	0.346
Female	4.53 ± 2.05		
City of work			
Inside Buraydah	4.89 ± 2.32	0.570	0.569
Outside Buraydah	4.59 ± 1.66		
Professional status^‡^			
General practitioner	4.67 ± 1.92	7.961	0.019**
Resident	3.81 ± 1.97		
Specialist/consultant	5.44 ± 1.96		
Years of experience^‡^			
<5 years	4.98 ± 1.81	2.016	0.365
5–10 years	4.52 ± 2.08		
>10 years	4.45 ± 2.26		
Have you ever attended a course on the management of anaphylaxis?			
Yes	4.94 ± 2.26	0.831	0.406
No	4.61 ± 1.81		
Have you ever been trained in the management of anaphylaxis?			
Yes	5.06 ± 2.02	2.369	0.018**
No	4.09 ± 1.80		
Have you ever met a patient with anaphylaxis?			
Yes	4.60 ± 2.04	0.868	0.386
No	4.89 ± 1.95		
Do you know where the nearest allergy clinic is in your area?			
Yes	4.79 ± 1.94	0.112	0.911
No	4.70 ± 2.04		
Do you keep epinephrine drugs in your department?			
Yes	4.74 ± 2.08	0.162	0.871
No	4.70 ± 1.56		
Does your primary healthcare center have a protocol to treat anaphylaxis?			
Yes	4.73 ± 1.99	0.324	0.746
No	4.75 ± 2.03		

In post hoc analysis (Table 5), it was revealed that there was a significant difference in the knowledge scores between residents and specialists/consultants (p=0.009).

**Table 5:** Multiple mean differences in knowledge scores in relation to the professional status Post hoc analysis was conducted using the Dunn-Bonferoni test. *The mean difference is significant at the 0.05 level.

(I) Professional group	(J) Professional group	Mean difference (I-J)	Std. error	Sig.	95% Confidence interval	
					Lower bound	Upper bound
General practitioner	Resident	0.85714	0.48505	0.239	-0.3209	2.0351
	Specialist/consultant	-0.77451	0.40870	0.182	-1.7671	0.2180
Resident	General practitioner	-0.85714	0.48505	0.239	-2.0351	0.3209
	Specialist/consultant	-1.63165^*^	0.53733	0.009	-2.9366	-0.3267
Specialist or consultant	General practitioner	0.77451	0.40870	0.182	-0.2180	1.7671
	Resident	1.63165^*^	0.53733	0.009	.3267	2.9366

## Discussion

Level of knowledge

The present study evaluated the knowledge and attitude of primary care physicians working in the Qassim region of Saudi Arabia, regarding anaphylaxis. The physicians' overall knowledge of this life-threatening allergy was inadequate. Based on the given criteria, nearly half (48.8%) were considered to have poor knowledge, 43% to have moderate knowledge, and only 8.3% to have a good knowledge level (mean score, 4.74 out of 10 points). This is consistent with the study of González-Díaz et al. where the overall approval percentage was only 28.7% [22]. In comparison to Saudi teachers, low levels were also seen in most of the teachers (85.3%); though their attitudes were mostly positive, their practices were also deemed poor (48.9%). However, in a study among UK parents, parental knowledge significantly improved after just a single visit to a pediatric allergy clinic [5]. Improvements were seen particularly in the knowledge of managing allergic reactions, allergen avoidance, and EpiPen practice. Allergic reactions among their children were also seen to decrease after the first visit. To be able to bridge the gaps, strategies to improve knowledge of anaphylaxis should be prioritized. Appropriate understanding of anaphylaxis is necessary to provide optimum care during life-threatening situations.

Significant factors affecting knowledge

Significant predictors for increased knowledge include being a consultant or specialist and being trained to manage anaphylaxis patients. This finding mirrored the study results of Sipahi Cimen and Sayili [23]. Healthcare professionals who received training and encountered anaphylaxis patients were more likely to exhibit better knowledge of epinephrine dosing, which was also consistent with the reports of Arga et al. [24]. Studies published in India also found variations in knowledge between each professional group, wherein nurses and nursing students were seen to have low levels of knowledge compared to other healthcare professionals and medical students [25,26]. However, AlHaddad et al. found no significant differences between the knowledge score and different job categories [19]. In our study, we noted that the differences in knowledge scores in relation to age, gender, city of work, and years of experience did not reach statistical significance (p>0.05).

Specific assessment of knowledge

The outcome of physicians' knowledge stemmed from the 10-item questionnaire on the basic facts of anaphylaxis. It is assumed that physicians' understanding of anaphylaxis basic facts was not ideal. For instance, only 19.8% understood the appropriate dose of epinephrine as an auto-injector. Their knowledge about the correct location for administering epinephrine in patients with anaphylaxis was also found to be low (27.3%); despite the fact that most physicians (90.1%) knew the first-line treatment for anaphylaxis, their understanding of the recommended intravenous dose did not sound adequate (31.4%). In addition, there seemed to be a lack of understanding about the duration of follow-up in cases of anaphylaxis (33.9%). In Riyadh, pediatric emergency physicians seemed to know basic anaphylaxis facts better [27]. For example, epinephrine was identified as the first-line treatment for anaphylaxis (80.2%), the intramuscular route for epinephrine was preferred by the majority (77.1%), and 73.7% would refer anaphylactic patients to an allergy specialist. Epinephrine seems to be the most prominent choice as the first-line treatment for anaphylaxis, which was proven in the literature [6,13,18,25,26].

Training and managing cases of anaphylaxis

Managing cases of anaphylaxis can significantly improve knowledge. In this study, more than half (53.7%) had the experience of managing cases of anaphylaxis. In the United States, 86% of the pediatricians indicated providing care for patients with food allergies, while in Turkey, only about one-third mentioned that they treated cases of anaphylaxis [6,18]. Furthermore, even though most of our study subjects (95.9%) felt that they needed more training or courses related to managing these cases, their actual attendance was a bit low (38.8%). Hence, it is necessary to encourage physicians to attend courses/trainings on the management of this life-threatening allergy.

Knowledge of epinephrine auto-injectors

More than 70% of our study population had heard of epinephrine auto-injectors, but the lack of this type of equipment in their workplace settings was visible. Only 19.8% reported to have it in their centers. In one of the previous studies published in Turkey, healthcare providers mentioned that epinephrine was available in their department; however, only 20.3% knew about epinephrine auto-injectors [18]. Another study from Turkey cited that healthcare workers' ability to use epinephrine auto-injectors was unreliable [24]. The authors suggest that the industry should keep on innovating to develop the ideal life-saving device.

Study limitations

In this study, there were also certain research limitations present. First, our sample size was small (n=121), which limits its generalizability. It could be interesting to see a bigger sample size that could provide a better overview of the study population's understanding of managing anaphylaxis patients. Second, many of our respondents were general practitioners; we may not be able to generalize pairwise comparisons with other professional groups due to the abnormal data distribution. Lastly, the study's cross-sectional nature makes it challenging to determine what factors led to the outcome.

## Conclusions

It was found that the knowledge among primary healthcare physicians regarding anaphylaxis was unsatisfactory. Consultants or specialists who attended training for managing anaphylaxis cases were likelier to demonstrate a better understanding of anaphylaxis than the rest of the primary care physicians. Physicians' lack of interest in the subject was evidently seen in this study. Although many had trained in managing anaphylaxis patients, continuous attendance at courses to update themselves seemed suboptimal. The gaps in the knowledge among physicians regarding anaphylaxis could lead to unwarranted patient outcomes. Hence, educating physicians about the management of anaphylaxis can enhance the efficiency of healthcare services.
